# Reduced spiral ganglion neuronal loss by adjunctive neurotrophin-3 in experimental pneumococcal meningitis

**DOI:** 10.1186/1742-2094-8-7

**Published:** 2011-01-24

**Authors:** Cornelia Demel, Tobias Hoegen, Armin Giese, Barbara Angele, Hans-Walter Pfister, Uwe Koedel, Matthias Klein

**Affiliations:** 1 Department of Neurology, Klinikum Grosshadern, Ludwig Maximilians University Munich, Marchioninistrasse 15, 81377 Munich, Germany; 2 Center of Neuropathology and Prion Research, Ludwig Maximilans University Munich, Feodor-Lynen-Strasse 23, 81377 Munich, Germany

## Abstract

**Background:**

Hearing loss is a frequent long-term complication of pneumococcal meningitis (PM). Its main pathological correlate is damage to the organ of Corti and loss of spiral ganglion neurons. The only current treatment option is cochlear implants which require surviving neurons. Here, we investigated the impact of systemically applied neurotrophin-3 (NT-3) on long-term hearing loss and the survival of neurons.

**Methods:**

Eighteen hours after infection with *S. pneumoniae*, C57BL/6 mice were treated with a combination of ceftriaxone with NT-3 or dexamethasone or placebo. Hearing, cochlear damage, and brain damage were assessed by audiometry and histology.

**Results:**

The main findings from immunohistochemical visualization of neurotrophins (NT-3, BDNF) and their receptors (TrkB, TrkC, and p75) in the cochlea were (i) enhanced staining for the cell survival-promoting receptor TrkB and (ii) increased NT-3 staining in NT-3 treated mice, showing that systemically applied NT-3 reaches the cochlea. The major effects of adjunctive NT-3 treatment were (i) a reduction of meningitis-induced hearing impairment and (ii) a reduction of spiral ganglion neuronal loss. The efficacy of NT-3 therapy was comparable to that of dexamethasone.

**Conclusion:**

Systemically applied NT-3 might be an interesting candidate to improve hearing outcome after pneumococcal meningitis.

## Introduction

Despite calculated antibacterial therapy and supportive intensive care, bacterial meningitis remains a very serious infectious disease with approximately 1.2 million cases per year worldwide causing 135.000 deaths [1]. The most frequent causative pathogen in adults is *Streptococcus *(S.) *pneumoniae*, leading to death in 15-25% of cases [2]. Up to 50% of survivors suffer from long-term sequelae. Sensorineural hearing loss is one of the most prevalent acute and long-term complications. It can manifest as uni- or bilateral, and as mild to severe hearing impairment, affecting one fourth of survivors [2-4].

In the course of acute bacterial meningitis, infection spreads most likely from the subarachnoid space through the cochlear aqueduct, reaching the perilymphatic spaces and causing suppurative labyrinthitis [5-7]. A massive immune response directed against the bacteria leads to collateral damage of the cochlea's own tissue [8,9]. Histopathological findings show a blood-labyrinth barrier disruption, damage to the organ of Corti, and destruction of spiral ganglion cells in the acute phase [2,10-14]. Loss of spiral ganglion neuronal cells as well as fibrocytic reorganisation of the perilymphatic spaces, leading to labyrinthitis ossificans, are noted as long-term residues [5,15]. In addition to the inflammatory response, direct bacterial toxicity may be a factor driving cochlear damage in meningitis. E.g., intracisternal inoculation of strains deficient in pneumolysin production is associated with significantly lower cochlear injury [16].

Currently, the only treatment option for severe long term hearing loss in pneumococcal meningitis is surgical implantation of a cochlear implant [17,18]. The efficacy of cochlear implant surgery depends on multiple factors, which include cognitive measures, proper surgical insertion, and the length of time after onset of deafness [19-21]. Moreover, at least a critical number of neurons seem necessary for proper functioning of a cochlear implant. This is underscored by the finding of a recent study that all patients who benefited from cochlear implantation had at least some spiral ganglion neuronal cells (SGCs) remaining (whereas peripheral nerve fibres or hair cells were completely absent in most patients) [22]. Bacterial meningitis can result in a dramatic reduction in the number of SGCs [6]. Additionally, the GC population was found to decline (further) over time after meningitis [23,24]. Therefore, therapeutic approaches to protect neurons from cell death during and after meningitis might have the potential to improve the efficacy of cochlear implants.

The neurotrophins are a group of proteins that induce survival, differentiation and neurite outgrowth. Two such neuroprotective agents, neurotrophin-3 (NT-3) and brain-derived neurotrophic factor (BDNF), play a significant role in the cochlea [25]. They are released by cochlear sensory cells and act via tyrosine kinase receptors (TrkB and TrkC) and p75-receptors that are expressed by spiral ganglion neuronal cells [26,27]. Studies using knock-out mice lacking either BDNF, NT-3, or both neurotrophins have demonstrated that both neurotrophic agents play a crucial role in the development and maintenance of spiral ganglion neuronal cells [28,29]. Administration of neurotrophin via mini-osmotic pumps, drug-eluting cochlear implants or viral vectors in animal studies have shown promising results in protecting auditory neurons and even in partly counteracting hearing loss [17,30-33]. In this study, we investigated the impact of systemically administered NT-3 on the preservation of cochlear neurons and hearing loss. Furthermore, we investigated the impact of NT-3 on neurologic outcome in a mouse model of experimental pneumococcal meningitis. Adjunctive therapy with NT-3 was compared to adjunctive dexamethasone treatment which has been recommended for adjunctive treatment of pneumococcal meningitis in adults [34,35].

## Methods

### Mouse model of pneumococcal meningitis

A well-characterized mouse model of pneumococcal meningitis was used in this study [36]. The model has been developed in C57BL/6 mice which are widely used for studies of cerebral infection and for studies on acquired hearing loss. C57BL/6 mice, however, carry the AHL locus for genetic hearing loss (*Cdh23*^*ahl*^) [37]. All animals were of the same age (4-6 weeks), at a time point months before age-related hearing loss occurs in these mice [38]. In brief, meningitis was induced by transcutaneous intracisternal injection of 15 μl of a bacterial suspension containing 10^6 ^colony forming units (cfu)/ml of *S. pneumoniae (SP) *D39 or placebo under short-term anaesthesia with enflurane. Mice were weighed, put into cages, allowed to wake up and fed with a standard diet and water ad libitum. Eighteen hours after infection, when all mice showed clinical signs of meningitis, intraperitoneal therapy with ceftriaxone was begun and continued for a total of 4 days. Furthermore, animals received adjunctive therapy or placebo (see experimental groups). Two weeks after infection, mice were deeply anaesthetized with ketamine and xylazine (SIGMA, St. Louis, MO, USA) and perfused transcardially with 15 ml of ice-cold phosphate buffered saline (PBS) containing 10 U/ml heparin. Temporal bones and brains were dissected, fixed in 4% formalin, decalcified in PBS containing 10% EDTA (SIGMA, St. Louis, MO, USA), and embedded in paraffin. All animal experiments were approved by the government of Upper Bavaria, Germany.

### Experimental groups in the mouse model

The following groups were investigated: (i) mice intracisternally injected with 15 μl sterile phosphate-buffered saline (PBS) (CON; n = 8); (ii) mice intracisternally injected with SP, treated with 100 mg/kg ceftriaxone (Roche, Grenzach-Wyhlen, Germany) daily for 4 days and 0.5 ml isotonic saline every 2nd day for 2 weeks (PLC, n = 16); (iii) mice intracisternally injected with SP, treated with 100 mg/kg ceftriaxone daily for 4 days and 0.5 mg/kg dexamethasone (Merck, Darmstadt, Germany) every 8 h for a total of 4 days (DEX, n = 12); (iv) mice intracisternally injected with SP, treated with 100 mg/kg ceftriaxone daily for 4 days and 25 μg/kg Neurotrophin-3 (R&D Systems, Wiesbaden, Germany) every 2nd day for 2 weeks (NT-3, n = 11). Infected placebo-treated mice (PLC) were compared with uninfected mice (CON), showing successful infection and development of meningitis and meningitis-associated hearing loss. To detect a possible adjunctive treatment effect, animals who received adjunctive therapy with dexamethasone (DEX) or neurotrophin-3 (NT-3) were compared with placebo-treated mice (PLC) and with each other.

### Clinical assessment of mice

Animals were investigated clinically before infection, 18 h, 42 h, and 2 weeks after infection. Clinical scores (CS) were determined as previously described reaching 0 points if there were no clinically noticeable signs of disease and 12 points if the animal died [39]. In brief, the following criteria were assessed: (i) beam balancing, (ii) postural reflexes, (iii) piloerection, (iv) epileptic seizures, and (v) level of consciousness. For determination of explorative activity, each mouse was put in the middle of a 42 × 42 cm box divided into 9 squares and allowed to explore the box for two minutes. The number of squares which the mouse passed through within the two minutes time interval was counted.

### Determination of hearing

Hearing was determined by auditory brain-stem responses (ABRs) at the end of the experiment. Mice were anaesthetized intraperitoneally with 100 mg/kg ketamine and 5 mg/kg xylazine. Needle electrodes were placed over each mastoid (negative pole), the vertex (positive pole), and the neck (reference). Impedances were controlled to be below 5 kΩ. Square-wave click impulses (duration: 100 ms; frequency: 20Hz) and tone bursts of 1 and 10 kHz (duration: 4 ms; frequency: 23.4 Hz) were delivered by earphones (E-A-RTONE3A, Aearo Company, Indianapolis, IN, USA). ABRs were amplified (x250.000), band-pass filtered (150-10,000Hz), and averaged (n = 1000) using a Neuroscreen Plus (Jaeger-Toennies, Freiburg, Germany). To determine the hearing threshold, we started with an impulse of 105 dB Sound Pressure Level (SPL) and reduced the intensity in 5 dB SPL steps. The lowest stimulus intensity that elicited visual ABRs was considered to be the hearing threshold. If a response could not be elicited at 105 dB SPL, stimulus intensities of up to 130 dB SPL were tested.

### Histologic assessment of cochleae

For histological analysis, midmodiolar sections (thickness 7 μm) of mouse temporal bone were deparaffinized, rehydrated, and stained with Mayer's haematoxylin and eosin (H&E; MERCK, Darmstadt, Germany). Sections were digitized using an Olympus BX51 microscope (Olympus Optical, Hamburg, Germany), connected to a camera (Moticam 5000, Motic Deutschland GmbH, Wetzlar, Germany). Two sections of each cochlea were analyzed and the mean was calculated for statistics.

For determination of the spiral ganglion neuronal density in the cochlea, the area of each spiral ganglion was measured (Image Tool Version 3.00, University of Texas Health Science Center, San Antonio, TX, USA) and morphologically intact spiral ganglion neurons (criteria: round cell body containing a nucleus and homogenous cytoplasm) were counted within this area.

A regular cochlear finding 2 weeks after pneumococcal meningitis is fibrocytic occlusion of the scala tympani, indicating early labyrinthitis ossificans. The degree of occlusion was evaluated by measurement of the occluded area of the basal turn of the tympanic scala (Image Tool Version 3.00, University of Texas Health Science Center, San Antonio, TX, USA). The occluded area was expressed as a percentage to the total area of the basal scala tympani.

The integrity of the organ of Corti was evaluated as (i) existing and intact, or (ii) absent or respectively damaged. The integrity of the organ of Corti was defined as intact inner and outer hair cells as well as an intact architecture of supporting cells.

### Immunohistochemical detection of neurotrophins and neurotrophin receptors

Cochleae were stained for NT-3 and BDNF as well as their receptors TrkB, TrkC (both inducing neuroprotective effects on neurons) and p75 (inducing apoptosis in neurons) using immunohistochemistry. Sections were deparaffinized and placed in a microwave for boiling in 10 mM citrate buffer (pH 6) for antigen retrieval. Endogenous peroxidases were blocked by incubation with 0.3% hydrogen peroxide in methanol (MERCK, Hohenbrunn, Germany). Sections were incubated with one of the following primary antibodies: (i) rabbit polyclonal anti-NT3 (1:500 in blocking solution, Alomone Labs Ltd, Jerusalem, Israel); (ii) rabbit polyclonal anti-BDNF (1:500 in blocking solution, Alomone Labs Ltd, Jerusalem, Israel); (iii) rabbit polyclonal anti-TrkB (1:500 in blocking solution, Novus Biologicals Inc., Littleton, CO, USA); (iv) goat monoclonal anti-mouse TrkC (1:200 in blocking solution, R&D Systems, Wiesbaden, Germany); (v) rabbit polyclonal anti-human p75 NTR (1:200 in blocking solution, Alomone Labs Ltd, Jerusalem, Israel) or (vi) blocking solution without primary antibody (negative control) in a humidified chamber overnight. Then, sections were incubated with secondary antibodies (biotinylated anti-rabbit IgG made in goat or anti-goat IgG made in horse according to the primary antibody used; both 1:200, Vector Labs, Burlingame, CA, USA). Binding of the secondary antibody was visualized with streptavidin horse-radish peroxidase (DAKO, Hamburg, Germany) and diaminobenzidine (Vector Labs, Burlingame, CA, USA), which yields a brown reaction product. Between all reaction steps, slides were rinsed with 0.1 M PBS (pH 7.4). Counterstaining was performed using Mayer's hematoxylin.

For assessment of immunohistochemical staining patterns, the most basal spiral ganglion was digitized. Pictures were blinded and printed on 20 × 30 cm color prints. Then, four reviewers (one professor of neuropathology, one assistant professor of neurology, and two residents of neurology with experience in neuropathology) independently grouped the printouts according to (i) homogeneity of the neuronal staining, (ii) intensity of the neuronal staining, and (iii) intensity of extracellular staining. A group was considered to be identified successfully if >80% of blinded slides were grouped together correctly.

### Histologic assessment in the brain

For histological analysis, 7-μm-thick hippocampal sections of mouse brains were used. For assessment of general brain pathology, sections were stained with Mayer's haematoxylin and eosin (H&E), Prussian blue (MERCK, Darmstadt, Germany) (detection of iron as a marker for residues of parenchymal hemorrhages), glial fibrillary acidic protein (GFAP, detection of astrocyte activation (rabbit GFAP polyclonal, 1:3000, DAKO, Hamburg, Germany), and neurofilament and amyloid precursor protein (APP, detection of axonal injury, Neurofilament monoclonal, 1:500, DAKO; APP A4 monoclonal, 1:100, Chemicon, Billerica, MA, USA; secondary antibody: swine anti-rabbit biotinylated, concentration 1:150, DAKO, Hamburg, Germany, visualization with streptavidin-horse radish peroxidase and diaminobenzidine). Activated (GFAP-positive) astrocytes (appearing with a round nucleus and more than two GFAP-positive processes) were counted within predetermined areas (cortex and hippocampus).

### Statistical analysis

All experimental procedures were performed in a blinded fashion. Data were analyzed with SYSTAT 9 (SPSS, Chicago, IL, USA) using a t-test for independent variables. P < 0.05 was considered significant. A Bonferroni correction was used to address the problem of multiple comparisons. Correlation analyses were performed according to Spearman. Data are displayed as mean ± standard deviation (SD).

## Results

### Clinical characteristics of meningitis

**Figure 1:**
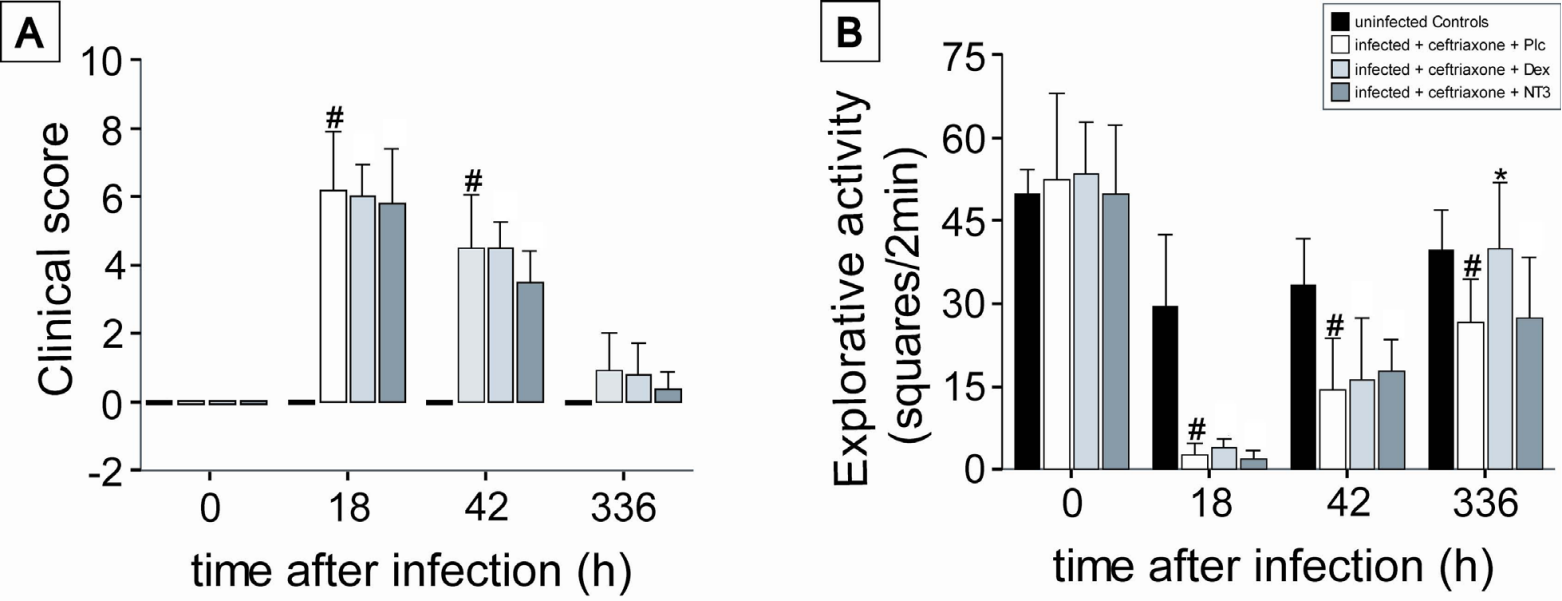
**Clinical Signs of Meningitis**. All infected mice showed clinical signs of disease 18 h after injection, reflected in (A) an elevated clinical score and (B) a tenfold reduced explorative activity compared to uninfected controls. Two weeks after infection, animals that had received adjunctive dexamethasone treatment showed explorative activity similar to uninfected controls whereas explorative activity in animals that had received placebo or adjunctive NT-3 was still reduced. (#) P < 0.05 as compared to uninfected controls, (*) p < 0.05 as compared to infected animals receiving ceftriaxone and placebo.

At 18 hours after infection, when treatment was begun, all infected mice had developed clinical signs of disease such as weight loss, an increased clinical score and reduced explorative activity. Within the next 24 hours following the start of the antibiotic therapy, the infected mice improved. By two weeks after infection, the clinical score of PLC mice almost completely resolved despite slight motor deficits and a reduced explorative activity (Figure 1). Mortality rates were similar in all groups (mortality PLC 4/16, NT-3 3/11, DEX 2/12); motor deficits and explorative activity did not differ between PLC, DEX, and NT-3.

Uninfected control animals (CON) that received intracisternal sterile PBS also showed a slight decrease of explorative activity, possibly due to a combination of a mild traumatic effect and/or habituation. However, their clinical scores were not altered, and they did not show signs of infection (such as pilo-erection), meningeal irritation or neurologic deficits.

### Impact of adjunctive NT3 and dexamethasone on hearing thresholds (HT)

Two weeks after infection, infected placebo-treated mice showed a significant elevation of hearing thresholds. An elevation of hearing thresholds was especially noted for high frequency hearing (10 kHz). This reflects the circumstance that cochlear damage was worst in the basal turn.

**Figure 2:**
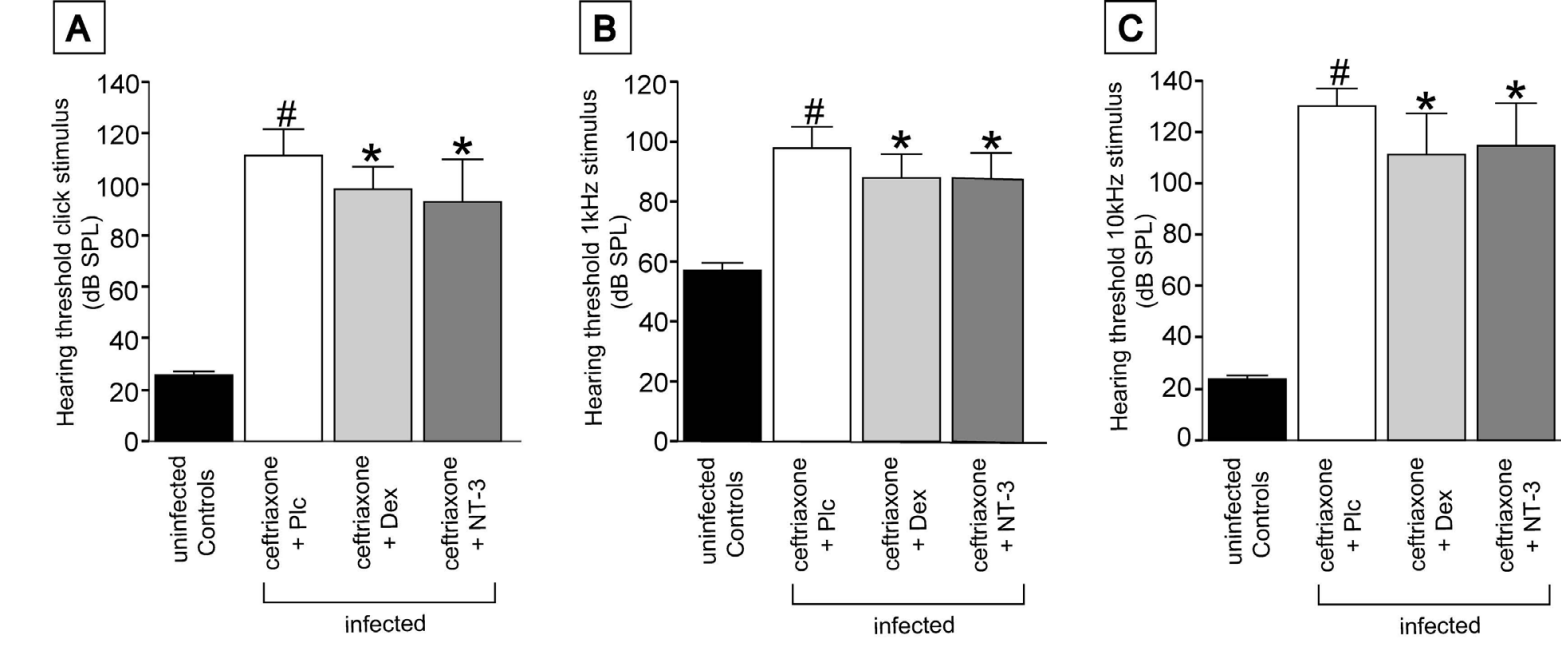
**Adjunctive dexamethason and NT-3 decrease hearing loss**. Mice were assessed for (A) click stimuli, (B) low frequency hearing (1 kHz stimuli), and (C) high frequency hearing (10 kHz stimuli). In infected animals, a significant increase of hearing thresholds was observed. The elevation of hearing thresholds was most severe for high frequency hearing. Adjunctive treatment with dexamethasone or NT-3 improved hearing significantly compared to mice treated with ceftriaxone plus placebo. There was no difference between dexamethasone and NT-3 treated animals. (#) P < 0.05 as compared to uninfected controls, (*) p < 0.05 as compared to infected animals receiving ceftriaxone and placebo.

Adjuvant therapy with NT-3 as well as dexamethasone mildly improved hearing outcome at two weeks after infection. (Figure 2)

### Impact of NT-3 and dexamethasone on cochlear spiral ganglion neuronal densities and cochlear occlusion

**Figure 3:**
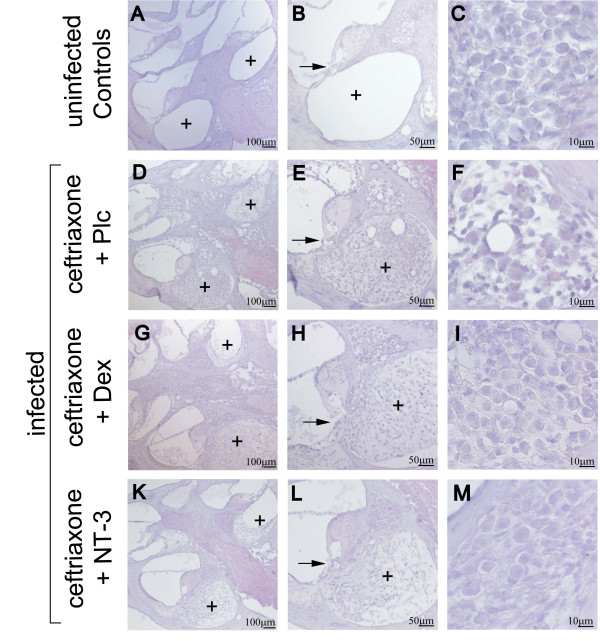
**Pathologic alterations in the cochlea**. (A-C) Cochleae of uninfected control animals showed an intact cochlear architecture (A, B) with a patent scala tympani (+) and (C) a dense population of neurons in the spiral ganglion. In infected animals that were treated with ceftriaxone and placebo, (D and E) dense fibrocytic occlusion of the scala tympani (+) was observed, (E) the organ of Corti (→) appeared destroyed, and (F) neuronal densities were decreased. Adjunctive treatment with (G, H, and I) dexamethasone or (K, L, and M) NT-3 did not lead to significant changes in (G, H, K, and L) fibrocytic cochlear occlusion or damage to hair cells but (I and M) both therapies rescued neuronal ganglion cells from death, reflected in less neuronal loss. The morphology of the cochlea did not differ between animals treated with dexamethasone or NT-3. Staining was performed with hematoxylin and eosin.

Meningitis led to damage of the organ of Corti, fibrocytic occlusion of the scala tympani, and loss of neurons in the basal spiral ganglion two weeks after infection (CON vs. PLC, Figure 3). The morphology of the organ of Corti was severely damaged or completely absent two weeks after infection with bacterial meningitis (CON vs. PLC: 0% vs. 54% (percentage of destroyed or completely absent organs vs. total number of investigated organs of Corti)). Adjuvant treatment, neither with NT-3 nor with dexamethasone, resulted in preservation of the organ of Corti (NT-3 29%, DEX 54% (destroyed or completely absent/total number of investigated organs of Corti)).

**Figure 4:**
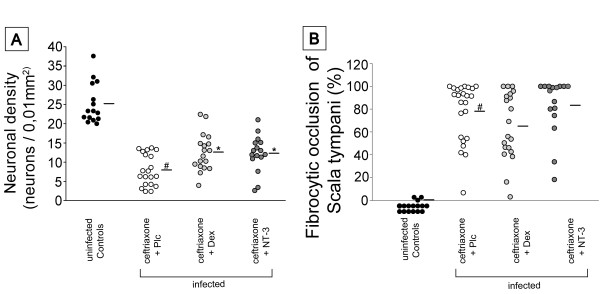
**Adjunctive dexamethasone and NT-3 decrease spiral ganglion neuronal loss**. (A) The density of intact neurons in the spiral ganglion of the cochlea was significantly reduced in PLC-treated animals two weeks after infection compared to uninfected control animals. Treatment with dexamethasone as well as NT-3 counteracted this decline and led to a decreased loss of neurons. (B) The scala tympani of cochleae of infected mice (PLC) were occluded with dense fibrocytic tissue which was not observed in healthy uninfected control animals (CON). Adjunctive dexamethasone or NT-3 did not have an impact on cochlear occlusion. (#) P < 0.05 as compared to uninfected controls, (*) p < 0.05 as compared to infected animals receiving ceftriaxone and placebo.

There was a significant decline of neuronal density in the basal spiral ganglion of infected mice (PLC) compared to uninfected controls (70%). Neuronal densities in the basal spiral ganglion correlated inversely with high frequency hearing thresholds (r^2^ = 0.51). Adjuvant therapy with NT-3 and dexamethasone led to a reduced loss of intact neurons in the spiral ganglion in comparison with the placebo group (Figure 4A).

Within infected mice, it was mainly the basal scala tympani that was obliterated with loose connective tissue. The mean occluded area in infected and placebo-treated animals (PLC) was 75.1% ± 27.52%. In contrast, the scala media (endolymphatic space) and the scala vestibuli did not show occlusions on a regular basis. There was no significant difference in regard to occlusion among the infected groups (Figure 4B). In summary, our data suggest that the hearing protective effects of both NT-3 and dexamethasone are mediated by a reduced loss of spiral ganglion neuronal cells.

### Neurotrophins and their receptors in the spiral ganglion

Two weeks after infection, there was a reduced number of intact neurons in the basal spiral ganglion of infected mice, which were more heterogeneously stained for neurotrophin NT-3 and BDNF than those of healthy control animals. All NT-3-treated mice showed an additional diffuse staining for NT-3 in the basal spiral ganglion that was not detected in any other group, indicating that systemically applied NT-3 reached the cochlea.

**Figure 5:**
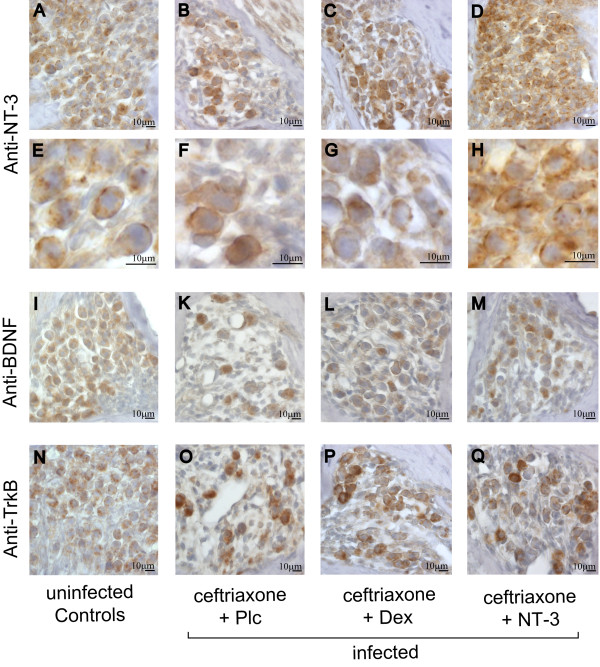
**Systemically applied NT-3 reaches the cochlea**. The number of intact neurons expressing (A-H) NT-3, (I-M) BDNF and (N-Q) TrkB homogeneously was higher in (A,E,I, and N) uninfected controls in contrast to infected mice ((B,F,K, and O) placebo-treated; (C,G,L, and P) dexamethasone-treated; (D,H,M, and Q) NT-3-treated). Only (D,H) NT-3-treated mice showed increased staining for NT-3, indicating that systemically applied NT-3 reached the spiral ganglion. (For staining details and qualitative blinded assessment of pictures see text).

Whereas uninfected controls stained homogeneously for TrkB, a more intense staining of most neurons was found in infected animals. This finding could possibly reflect an up-regulation of TrkB to increase the neuron's opportunity to survive a situation with decreased neurotrophin levels (Figure 5). The p75-positive neurons of uninfected controls and infected mice showed no difference in staining pattern; in addition, staining patterns for TrkC and BDNF were not altered between the groups (not shown).

### Morphological changes in the cortex and hippocampus

**Figure 6:**
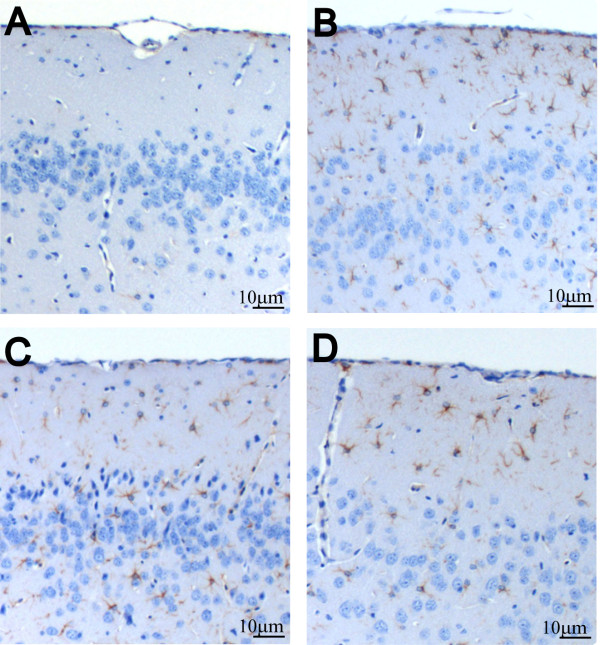
**Astrocyte activation after meningitis**. GFAP-positive astrocytes were not seen (A) in brains of uninfected control mice. In contrast, (B-D) in brains of infected mice, a high density of GFAP-positive astrocytes in the cortex reaching far into the superficial layer was observed. (B) Compared to infected placebo-treated animals, this was not altered by adjunctive treatment with (C) dexamethasone or (D) NT-3. GFAP staining (brown).

An elevated number of GFAP-positive astrocytes was found in the cortex of infected mice (PLC), reflecting activation of astrocytes as an indicator of former brain damage (CON vs. PLC: 12.7 ± 11.2 astrocytes/mm^2 ^vs. 88.9 ± 57.5 astrocytes/mm^2^, p = 0.003). There were no significant differences between the various treatment groups (NT-3 55.5 ± 28.3 astrocytes/mm^2^, DEX 116.3 ± 51.5 astrocytes/mm^2^) (Figure 6). Signs of previous parenchymal bleeding were absent (negative staining for iron). We also did not find pathological changes suggestive of necrosis or axonal injury in hippocampal or cortical areas two weeks after infection (data not shown).

## Discussion

In this study, a mouse model of pneumococcal meningitis was used that featured all typical clinical and histomorphological findings of meningitis-associated labyrinthitis and hearing loss to study the impact of adjunctive neurotrophins on cochlear damage. We demonstrated that (i) intraperitoneally applied NT-3 reached the cochlea, (ii) adjunctive NT-3 treatment led to a significant decrease of spiral ganglion neuronal damage and hearing loss, (iii) the effect of NT-3 therapy was comparable to the benefit of adjunctive therapy with dexamethasone, and (iv) changes in brain pathology could not be observed after treatment with NT-3.

Two weeks after infection we observed meningitis-associated morphological changes in the cochlea, namely fibrocytic occlusion of the scala tympani, damage to and often loss of the organ of Corti, as well as a significant decline of neurons in the spiral ganglion. The morphological alterations dominated in the basal turn of the cochlea, where high frequency hearing is localized. This was associated with pronounced high frequency hearing impairment as measured with auditory brainstem responses. These findings are similar to previous experimental observations and data from humans with bacterial meningitis, suggesting a spread of the infection from the brain to the inner ear via the cochlear aqueduct that enters the basal turn of the scala tympani [2,23].

Our immunohistochemical findings indicate that intraperitoneally applied NT-3 actually reached the area of interest in the cochlea. A previously published study described findings consistent with our study: After systemic application of NT-3 (via a subcutaneous Alzet micro-osmotic pump) neuroprotective effects were noticed in the dorsal root ganglia [40]. This demonstrates that NT-3 can pass the blood-labyrinth-barrier (in contrast to BDNF that has a larger molecular size), and this led us to test the effect of NT-3 in our mouse model of meningitis-associated hearing loss [41]. Another possible circumstance that could have facilitated the passage of NT-3 into the cochlea in our model is disruption of the blood-labyrinth barrier that occurs during the course of acute bacterial meningitis [5]. Importantly, systemic application of NT-3 does not require specific equipment as is the case for neurotrophins that are applied locally via mini-osmotic pumps, viral vectors or drug-eluting electrodes; and it does not bear the risk of local infection [42].

Adjuvant NT-3 led to a reduced loss of neurons in the spiral ganglion and lowered hearing loss after meningitis. A reduction of spiral ganglion neuronal loss through adjunctive NT-3 has not been previously shown in bacterial meningitis-associated labyrinthitis. Our data is strengthened by a recent study that demonstrated that NT-3 can preserve cochlear spiral ganglion neurons after ototoxic hair cell damage when locally applied [18]. Hair cell damage is a predominant correlate of long-term meningitis-associated hearing loss and was also present in our model. Possibly this contributes to the loss of neurons in the ganglion after meningitis through reduced depolarization of cells followed by neuronal inactivity and neuronal death [12,43]. Furthermore, a decline of hair cells could lead to a reduced supply of BDNF and NT-3, necessary for neuronal survival [44]. This might explain the neuroprotective effect of exogenous NT-3 administration after meningitis. Protection of neurons in the spiral ganglion after meningitis is important, since a critical number of surviving neurons are a prerequisite for the functioning of cochlear implants, which currently represents the only available treatment option for sensorineural hearing loss. Furthermore, NT-3 not only reduced neuronal degeneration but even led to a reduction of hearing loss. Therefore, systemic adjuvant therapy with NT-3 might be an option for co-treatment of patients with pneumococcal meningitis.

Systemic administration of neurotrophins can lead to side effects. However the complete range of side effects cannot be predicted at present, because of a lack of experience and a lack of knowledge of the role of neurotrophins in certain systemic diseases: Neurotrophins have been shown to be present constitutively in the blood, and to be elevated in certain pathologic conditions. E.g., high levels of neurotrophins have been reported in patients with rheumatoid arthritis, systemic lupus erythaematosis, allergic disease, or asthma as well as type 1 diabetes and metabolic syndrome [45-48]. *In vitro*, an elevation of neurotrophins in response to proinflammatory cytokines has been found, suggesting a possible link between neurotrophins and inflammatory processes [49]. So far, it is suspected that systemic administration of TrkB agonists can lead to anorexia and weight loss, and systemic therapy with nerve growth factor (NGF) has been shown to evoke myalgias and arthralgias [50,51]. To date, systemic neurotrophin-3 has only been applied to humans in small case series with limited side effects [52]. Due to this limited experience and the fact that clinical studies on adjunctive NT-3 after meningitis are lacking, systemic NT-3 cannot be recommended for the use in humans with meningitis at this time.

With respect to changes in the brain, a benefit of adjunctive therapy with NT-3 or dexamethasone could not be observed: activation of astrocytes, for instance, reflected in GFAP-positive astrocytes, was not altered by adjunctive therapy. Gliosis occurs in response to all forms of CNS injury or disease. The functions of reactive astrocytes are not well understood and both harmful and beneficial activities have been attributed to these cells. However, we could not detect severe brain damage two weeks after infection in our model. Signs of parenchymal bleeding or axonal injury, which are typical findings of severe experimental murine meningitis, have not been observed as documented by negative iron staining of the brain [53,54]. The most likely explanation is the early start of antibiotic therapy at 18 hours after infection. This time frame was chosen because the focus of the study was on a possible treatment effect on hearing loss, requiring less-than-complete damage of the inner ear as observed when treatment is begun early (18 h after infection). A later start of antibiotic therapy in mice (e.g. 24 h after infection) results in an irreversible hearing impairment [55]. In addition, brain pathology is then more pronounced (e.g. damage of neurons, vasculitis, and ischemia) and mortality is higher [53,54]. Therefore, a possible effect of adjunctive therapy on pathological changes in the brain might have been missed in the current study.

The impact of long-term NT-3 therapy on long-term hearing loss was compared not only with placebo-treated animals but also with animals that received adjunctive therapy with dexamethasone, which has been recommended as the standard adjunctive therapy of choice in patients with pneumococcal meningitis [34,56]. Here, therapy with NT-3 was of clear benefit in comparison with adjunctive placebo and similar in effectiveness to adjunctive dexamethasone. Just recently, however, the benefit of dexamethasone in pneumococcal meningitis has been questioned by a large meta-analysis of individual patient data [57]. Currently, it is unclear how this observation will affect future treatment guidelines. This highlights the need for further adjunctive therapeutic approaches. At present, dexamethasone is not recommended in patients with immunosuppression and contraindications to corticosteroids. In such patients, adjunctive NT-3 therapy might be an interesting alternative to dexamethasone. At present, data on NT-3 therapy in pneumococcal meningitis is still experimental and experience on possible side effects of systemic NT-3 treatment as well as the high costs of NT-3 need to be kept in mind.

In conclusion, systemically applied NT-3 reached the spiral ganglion, leading to a reduced loss of spiral ganglion neurons. Furthermore, this neuroprotective agent had a positive impact on hearing thresholds. Compared with the currently established adjuvant therapy with dexamethasone, NT-3 showed similar effects. Systemic NT-3 could be a valid alternative to reduce damage to spiral ganglion neuronal cells and hearing loss after bacterial meningitis.

## List of abbreviations

ABR: auditory brain-stem responses; AHL: age-related hearing loss; APP: amyloid precursor protein; BDNF: brain derived neurotrophic factor; cfu: colony forming units; Con: controls; CS: clinical score; Dex: dexamethasone; EDTA: ethylenediaminetetraacetic acid; GC: ganglion cell; GFAP: glial fibrillary acidic protein; H&E: haematoxylin and eosin; HT: hearing threshold; IgG: Immunoglobulin G; NT-3: Neurotrophin-3; PBS: phosphate buffered saline; Plc: placebo; PM: pneumococcal meningitis; SD: standard deviation; SGC: spiral ganglion cell; SP: Streptococcus pneumonia; SPL: sound pressure level; TrkB: tyrosine kinase receptor B; TrkC: tyrosine kinase receptor C

## Competing interests

The authors declare that they have no competing interests.

## Authors' contributions

The work presented here was carried out in collaboration between all authors. Experimental procedures were carried out by MK and CD with technical assistance of BA. Histological assessment of slides was performed by CD, MK, UK, AG, and TH. The manuscript was drafted by CD and MK and discussed and edited by all co-authors. All authors have read and approved the final version of the manuscript.
